# Changes in HER3 expression profiles between primary and recurrent gynecological cancers

**DOI:** 10.1186/s12935-022-02844-z

**Published:** 2023-02-03

**Authors:** Yuki Kojima, Kazuki Sudo, Hiroshi Yoshida, Shu Yazaki, Momoko Tokura, Chiharu Mizoguchi, Hitomi S. Okuma, Shosuke Kita, Kasumi Yamamoto, Tadaaki Nishikawa, Emi Noguchi, Tatsunori Shimoi, Yasuhito Tanase, Masaya Uno, Mitsuya Ishikawa, Tomoyasu Kato, Kumiko Koyama, Maki Kobayashi, Tomoya Kakegawa, Yasuhiro Fujiwara, Kan Yonemori

**Affiliations:** 1 Department of Medical Oncology, National Cancer Center Hospital, 5-1-1, Tsukiji, Chuo-Ku, Tokyo, 104-0045 Japan; 2 Department of Diagnostic Pathology, National Cancer Center Hospital, 5-1-1, Tsukiji, Chuo-Ku, Tokyo, 104-0045 Japan; 3 Department of Gynecology, National Cancer Center Hospital, 5-1-1, Tsukiji, Chuo-Ku, Tokyo, 104-0045 Japan; 4 Translational Science Department I, Daiichi Sankyo Co., Ltd., 1-2-58, Hiromachi, Shinagawa-Ku, Tokyo, 140-8710 Japan; 5 Translational Research Department, Daiichi Sankyo RD Novare Co., Ltd., 1-16-13, Kitakasai, Edogawa-Ku, Tokyo, 134-8630 Japan

**Keywords:** HER3, Ovarian cancer, Endometrial cancer, Cervical cancer; biomarker

## Abstract

**Background:**

Human epidermal growth factor receptor-3 (HER3) is a member of the epidermal growth factor receptor family of receptor tyrosine kinases, and its overexpression is associated with inferior prognosis in several cancers. However, it is unclear whether HER3 expression status changes in tumor tissue at recurrence. Therefore, this study aimed to evaluate the changes in HER3 expression between primary and recurrent status in gynecological cancers.

**Methods:**

This retrospective study used matched-pair tissues of gynecological cancer patients at initial diagnosis and at recurrence. Immunohistochemical (IHC) scores of 3 + or 2 + were termed “HER3-high”, while IHC scores of 1 + or 0 were designated as “HER3-low/zero”.

**Results:**

A total of 86 patients (40 with ovarian cancers, 32 with endometrial cancers, and 14 with cervical cancers) were included in this study. In ovarian cancer, 67.5% and 80.0% of the patients received a HER3-high at initial and recurrent diagnosis, respectively. The H-score was significantly increased at recurrence (p = 0.004). The proportion of HER3-high endometrial cancer patients increased from 46.9% at initial diagnosis to 68.8% at recurrence, and the H-score tended to increase at recurrence (p = 0.08). The fraction of HER3-high-rated cervical cancer patients remained unchanged at 85.7% both at initial and recurrent diagnosis. The discordance rate of HER3 expression detection in initial and recurrent diagnosis samples was 27.5%, 53.1%, and 14.3% for ovarian, endometrial, and cervical cancers, respectively. Ovarian and endometrial cancers with a HER3-high recurrent score tended to show shorter median survival time than those with a HER3-low/zero recurrent rating.

**Conclusion:**

Our findings suggest that, in main types of gynecological cancers, the proportion of patients having a HER3-high score increased from initial to recurrent diagnosis.

**Supplementary Information:**

The online version contains supplementary material available at 10.1186/s12935-022-02844-z.

## Background

Human epidermal growth factor receptor-3 (HER3) is a pseudokinase member of the epidermal growth factor receptor (EGFR) family. It heterodimerizes with receptor tyrosine kinases to activate oncogenic signaling via the the phosphatidylinositol 3-kinase/protein kinase B pathway [1, 2]. Overexpression of HER3 is observed in several cancers [3–9] and is associated with inferior prognosis [10–15]. In addition, several reports have suggested a pathogenic role for HER3 in mechanisms underlying primary or acquired resistance to EGFR inhibitors [16–18] and anti-HER2 therapies [19]. Therefore, HER3 is a promising therapeutic target, and several HER3 targeting drugs are currently being studied [20–26].

In gynecological cancers, the incidence of HER3 overexpression was reported to be 41.3–67.5% for ovarian cancer [5, 27, 28], 30% for endometrial cancer [29], and 31.0–74.7% for cervical cancer [15, 30–32]. However, most studies have only reported the incidence of HER3 expression at initial diagnosis, while that at recurrent diagnosis was not evaluated. In this study, we evaluated HER3 expression in matched-paired specimens taken from gynecological cancer patients at initial and recurrent diagnosis to evaluate HER3 as a promising biomarker for overcoming treatment failure and improving patient outcomes.

## Materials and methods

### Study population

This study included gynecological cancer patients with matched-pair tissue samples taken at initial diagnosis and at recurrence between 1999 and 2019 at the National Cancer Center Hospital, Japan. Patients with unavailable or insufficient tumor tissue were excluded. Finally, 86 patients were included (40 with ovarian, 32 with endometrial, and 14 with cervical cancers; Additional file 1: Table S1, Additional file 2: Table S2, Additional file 3: Table S3). We retrospectively collected the following clinical and pathological data: age, histology, stage as defined by the International Federation of Gynecology and Obstetrics in 2008 [33], lymph node metastasis, adjuvant treatment, and postoperative survival time.

The study was approved by the Institutional Review Board of the National Cancer Center, Tokyo, Japan (Approval Number: 2020–003). Written informed consent was waived because of the retrospective design.

### Immunohistochemical staining and evaluation

We performed immunohistochemical (IHC) staining for HER3 as previously described [34, 35]. Briefly, sections of each sample were deparaffinized and antigen retrieval was performed at high pH (PT Link machine, Dako). The sections were then stained with a rabbit monoclonal antibody against HER3/ErbB3 (1:59 dilution; clone D22C5, Cell Signaling Technology Inc., Danvers, MA, USA) using the Dako autostainer Link48 (Dako, CA, USA) and EnVision Flex Mini Kit (Dako), according to the manufacturer’s instructions. Hematoxylin was used as a nuclear counterstain. HER3-high was defined as an IHC score of 3 + or 2 + , and HER3-low/zero was defined as an IHC score of 1 + or 0 in line with the HER2 testing guidelines for gastroesophageal cancer [36]. The H-score (range, 0–300) was calculated using the following formula: 3X + 2Y + Z, where X, Y, and Z are the percentages of tumor cells showing strong, moderate, and weak staining intensities, respectively [37] (Additional file 1: Table S1A–D). The specificity of this HER3 IHC staining method was validated by confirming no signal detected with an isotype control antibody. Both positive and negative cell line control slides were also incorporated into HER3 staining to verify the specificity at every batch of staining.

### Statistical analysis

Differential HER3 expression between initial diagnosis and at recurrence was evaluated using the following tests; the χ^2^ test for categorical variables and the Mann–Whitney U test for continuous variables. Overall survival (OS) was estimated using the Kaplan–Meier method. The log-rank test was used to compare survival between groups. OS was defined as the time from relapse to death from any cause. All tests were two-tailed, and the significance level was set at α = 0.05. All statistical analyses were performed using GraphPad Prism ver.8.0 (GraphPad Software, San Diego, California, USA).

## Results

### Changes in HER3 expression in ovarian cancer

**Fig. 1:**
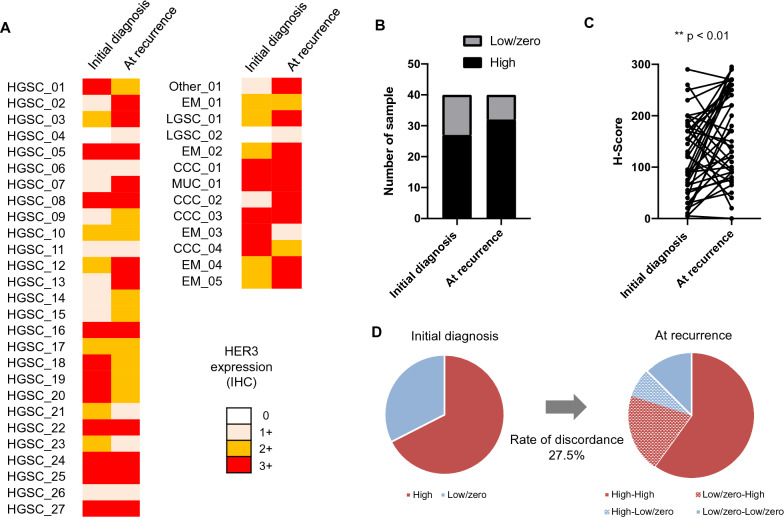
Changes in HER3 expression of ovarian cancer at initial diagnosis and at recurrence. **a** Individual sample cases are designated by columns and representative of 0, 1 + , 2 + , and 3 + staining patterns for HER3 expression. Left; initial diagnosis, right; at recurrence. HGSC; high-grade serous carcinoma, LGSC; low-grade serous carcinoma, EM; Endometrioid carcinoma, CCC; Clear cell carcinoma. **b** Proportion of HER3-high at initial diagnosis and at recurrence. **c** H-score of HER3 at initial onset and recurrence. **d** Discordance rates of HER3 assessment between initial diagnosis and at recurrence

HER3 expression IHC scores at initial diagnosis were ≥ 1 + in 95.0% of cases, while 67.5% (27 cases) showed scores ≥ 2 + (HER3-high; Fig. 1a and Additional file 4: Table S4). At recurrence, 80.0% (32 cases) had HER3-high scores (Fig. 1a, b). H-scores were significantly higher at recurrence (*p* = 0.0041, t-test; Fig. 1c); 27.5% of patients had altered HER3 designations (Fig. 1d). Of the 13 HER3-low/zero patients, 61.5% (eight cases) changed to HER3-high. Among the 27 HER3-high patients, 11.1% (three cases) changed to HER3-low/zero.

No significant differences were found for HER3-high scores at recurrence in any of the following: high-grade serous carcinomas *vs*. others (77.8% and 84.6%, respectively), platinum-sensitive recurrence *vs*. -resistant recurrence (79.3 and 81.8%, respectively), localized relapse *vs*. distant metastases (75.0 and 83.3%, respectively), and one type of prior therapy *vs*. two or more (78.5 and 83.3%, respectively; Additional file 7: Figure S2a–d). In samples from patients receiving two or more chemotherapeutic regimens, the HER3-high score changed significantly between initial diagnosis and at recurrence (p = 0.04; Additional file 7: Figure S2d).

### Changes in HER3 expression in endometrial cancer

**Fig. 2:**
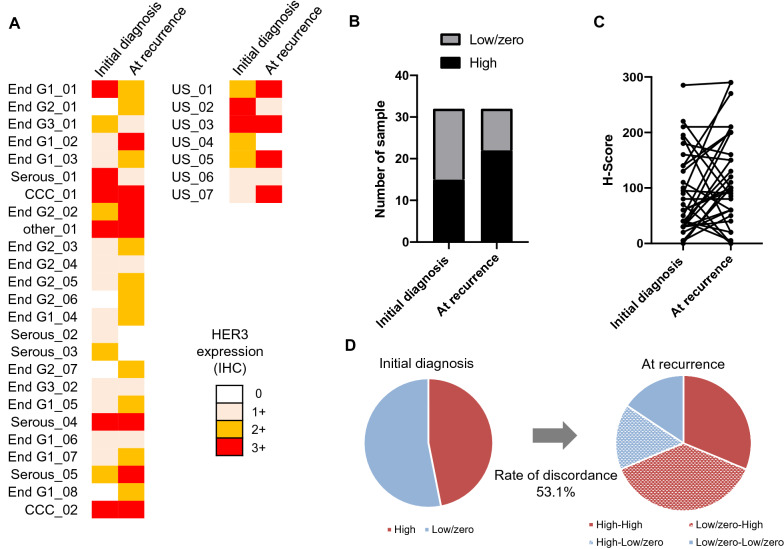
Changes in HER3 expression of endometrial cancer at initial diagnosis and at recurrence. **a** Individual sample cases are designated by columns and representative of 0, 1 + , 2 + , and 3 + staining patterns for HER3 expression. Left; initial diagnosis, right; at recurrence. End; endometrioid carcinoma, CCC; Clear cell carcinoma, US; uterine carcinosarcoma. **b** Proportion of HER3-high at initial diagnosis and at recurrence. **c** H-score of HER3 at initial diagnosis and at recurrence. **d** Discordance rates of HER3 assessment between initial diagnosis and at recurrence

Of the patient, 46.9% (15 cases) with endometrial cancer were HER3-high at initial diagnosis (Fig. 2a and Additional file 5: Table S5). At recurrence, 68.8% (22 cases) were HER3-high (Fig. 2a, b). H-scores tended to be lower at initial diagnosis (median, 65) than at recurrence (median, 100); however, this difference was not significant (*p* = 0.08, t-test; Fig. 2c). Changed HER3 score were identified in 53.1% (17 cases) of endometrial cases (Fig. 2d); 70.6% of patients (12 cases) changed from HER3-low/zero to HER3-high category, while 33.3% (five cases) changed from a HER3-high to a HER3-low/zero score.

Low-grade endometrial cancer was defined as G1 and G2, and high-grade endometrial cancer was denoted as others; 13.3% (two cases) of samples from low-grade endometrial cancers were HER3-high at initial diagnosis, which increased significantly to 86.7% (13 cases) at recurrence (p < 0.0001; Additional file 7: Figure S2e). No significant differences were found for HER3-high scores at recurrence in any of the following: localized relapse *vs*. distant metastases (66.7% and 72.7%, respectively) and non-chemotherapy *vs*. prior chemotherapy (61.5% and 73.7%, respectively) (Additional file 7: Figure S2f, g). In patient samples with distant metastases or prior chemotherapy, the HER3-high score increased significantly between initial diagnosis and at recurrence (p = 0.01 and p = 0.049, respectively; Additional file 7: Figure S2f, g).

### Changes in HER3 expression in cervical cancer

**Fig. 3:**
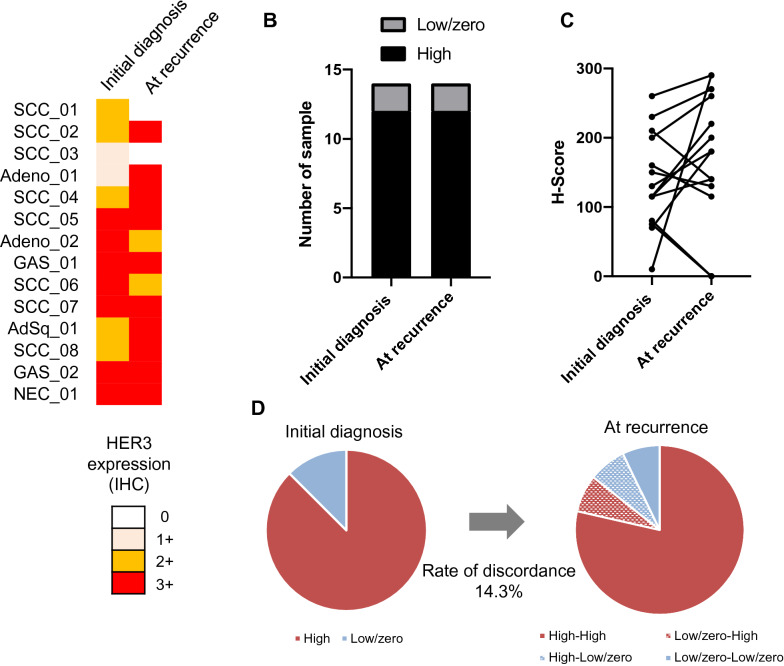
Changes in HER3 expression of cervical cancer at initial diagnosis and at recurrence. **a** Individual sample cases are designated by columns and representative of 0, 1 + , 2 + , and 3 + staining patterns for HER3 expression. Left; initial diagnosis, right; at recurrence. SCC; squamous cell carcinoma, Adeno; adenocarcinoma, AdSq; adenosquamous carcinoma, GAS; Adenocarcinoma, gastric-type, NEC; neuroendocrine carcinoma. **b** Proportion of HER3-high at initial diagnosis and at recurrence. **c** H-score of HER3 at initial onset and at recurrence. **d** Discordance rate of HER3 assessment between initial diagnosis and at recurrence

Of the patient population, 85.7% (12 patients) with cervical cancer were HER3-high at initial diagnosis (Fig. 3a and Additional file 6: Table S6); 85.7% (12 patients) were also HER3-high at recurrence (Fig. 3a, b). H-scores for HER3 showed a tendency to increase from initial diagnosis (median, 122.5) to recurrence (median,180), but this change was not significant (*p* = 0.19, t-test; Fig. 3c). HER3 expression status changed in 14.3% (two cases) of patients. One was HER3-high at diagnosis and HER3-low/zero at recurrence, while the other was HER3-low/zero at diagnosis and HER3-high at recurrence.

No significant differences were found for HER3-high scores at recurrence in any of the following: squamous *vs*. non-squamous (75.0% and 100.0%, respectively), localized relapse *vs*. distant metastases (87.5% and 83.3%, respectively), and non-radiotherapy *vs*. prior radiotherapy (83.3% and 87.5%, respectively).

### Association of HER3 expression with survival

**Fig. 4:**
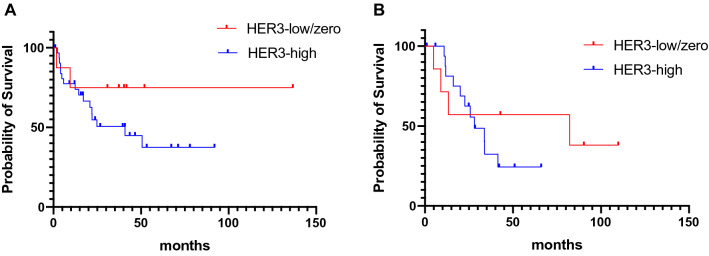
Kaplan‒Meier survival analysis of overall survival, including HER3 status at recurrence. **a** ovarian cancer and **b** endometrial cancer in patients with HER3-high vs. patients with HER3-low/zero at recurrence

We analyzed survival in patients with ovarian and endometrial cancers, excluding cervical cancer, which is much less common. Carcinosarcoma was excluded from the analysis among endometrial cancers because of its poor prognosis. At recurrence, the median survival time for HER3-high ovarian and endometrial cancers tended to show poorer OS than HER3-low/zero cancers. In ovarian cancers, HER3-low/zero cancers had an unreached OS (95% confidence interval (CI), 4.4 to 73.3 months), while HER3-high cancers had an OS of 40.9 months (95% CI 12.5 to 30.1 months), with a hazard ratio (HR) of 0.41 and a 95% CI of 0.13–1.24. In endometrial cancers, HER3-low/zero OS was 82.2 months. (95% CI 6.5 to 79.2 months), while HER3-high cancers had an OS of 28.1 months (95% CI 17.0 to 33.5 months), with an HR of 0.65 and 95% CI of 0.22–1.89 (Fig. 4a, b). In addition, we examined survival based on changes in HER3 expression status between diagnosis and at recurrence. For ovarian and uterine cancers, patients who became HER3-high tended to have a worse prognosis, while those who changed to HER3-low/zero tended to have an improved prognosis (Additional file 7: Figure S3a, b).

## Discussion

Here, we report the change in HER3 expression in gynecological tumor tissues of patients between initial and recurrent diagnosis. HER3 expression was elevated in all types of gynecologic cancers at recurrence (Table 1). While previous findings have reported HER3 expression at initial diagnosis [5, 27–32], to the best of our knowledge, this is the first study to demonstrate changes in HER3 expression among gynecologic cancers.

**Table 1:** Changes in HER3 expression at initial diagnosis and at recurrence

	HER3-high			H-score, median (IQR)		
	At initial diagnosis	At recurrence	*p*	At initial diagnosis	At recurrence	*p*
Ovarian cancer (N = 40)	27 (67.5%)	32 (80.0%)	0.20	120 (62.5–172.5)	160 (90–260)	**0.004**
Endometrial cancer (N = 32)	15 (46.9%)	22 (68.8%)	0.08	65 (30–140)	100 (60–170)	0.08
Cervical cancer (N = 14)	12 (85.7%)	12 (85.7%)	1.00	122.5 (88.75–190)	180 (132.5–250)	0.19
Total (N = 86)	54 (62.8%)	66 (76.7%)	**0.046**	97.5 (42.5–170)	135 (86.25–220)	** < 0.001**

Bold indicates significant difference

*IQR* interquartile range

HER3 heterodimerizes with HER1/HER2/HER4 and activates a signaling network that promotes tumor growth and metastasis [1, 2]. Molecularly targeted drugs against the HER family have been reported to increase HER3 expression [16, 18, 19, 38–40]. Janne et al. [20] reported the promising efficacy of partitumab derutecan in patients with EGFR-mutated non-small cell lung cancer who had developed resistance to EGFR inhibitors. In our study, HER3 expression at recurrence was equally high in patients treated with anti-cancer agents and those who remained untreated. In ovarian and endometrial cancer patients, HER3 expression at recurrence was significantly higher in those patients who received more types of anti-cancer therapy (Additional file 7: Figure S1d, f). These results suggest that HER3 expression at recurrence is independent of HER-targeted therapy.

The biological characteristics of cancer are known to change during progression and recurrence [41–43]. Possible causes for this modulation may include actual biological change, clonal selection due to treatment, sampling errors, and tumor heterogeneity [44]. Therefore, a re-biopsy for a patient at recurrence should be considered as an option before determining therapeutic regimens in clinical practice, especially in patients resistant to molecularly targeted drugs [45]. Our data show that HER3 expression is elevated at recurrence in all types of gynecologic cancers. In addition, the discordance rate of HER3-high scores in samples at initial diagnosis versus recurrence was 27.5% and 53.1% for ovarian and endometrial cancers, respectively (Figs. 1d, 2d). This result suggests that even in cancers with low HER3 expression at initial diagnosis, such as endometrial cancer, HER3 expression may still increase at recurrence. Thus, it is essential to evaluate recent samples rather than past tumor tissue when considering HER3 as a therapeutic target.

HER3 plays many roles in cancer cells, including mediating cell transformation and tumor malignancy [46, 47]. Cancer patients with high HER3 expression are reported to have poorer prognoses in several cancer types [10–15]. Our results indicate that increased HER3 expression at recurrence may be associated with an inferior prognosis (Fig. 4a, b). Thus, therapeutics targeting HER3 may improve the prognosis for patients with recurrent gynecological cancers.

This study has a few limitations. First, our report is based on a relatively small number of patient samples, which were all from a single institution. Second, although we did find increased HER3 expression in samples obtained at recurrence, we did not focus on the mechanisms underlying this increase. One possible reason for this observed regulation of HER3 expression may be clonal selection occurring during tumor progression. Other possible causes may include the postoperative treatment regimen, the site of recurrence, and the time to recurrence. Third, analyses of the association between changes in HER3 expression and survival have not been adjusted for prognostic factors or treatment modalities. Although the association with survival should be evaluated after adjusting for each factor, the small sample size in this study made evaluation difficult. Further evaluation in a larger population is needed to assess the association between changes in HER3 expression and survival. Finally, potential sampling restrictions may have affected our results since most initial diagnosis samples were surgical specimens, while most samples at recurrence were from biopsies.

## Conclusion

Our findings suggest that HER3 expression may be elevated at recurrence in patients with main types of gynecological cancers. Our data lend further support to the notion of HER3-targeted therapies being promising options for overcoming treatment resistance and improving outcomes in patients with gynecological cancers.

## Supplementary Information

**Additional file 1: Table S1.** Patient characteristics of ovarian cancer

**Additional file 2: Table S2.** Patient characteristics of endometrial cancer

**Additional file 3: Table S3.** Patient characteristics of cervical cancer

**Additional file 4: Table S4.** HER3 expression in ovarian cancer at initial diagnosis

**Additional file 5: Table S5.** HER3 expression in endometrial cancer at initial diagnosis

**Additional file 6: Table S6.** HER3 expression in cervical cancer at initial diagnosis

**Additional file 7.** Supplemental Figs. 1–3. **Supplemental Fig. 1**. Histological findings of HER3 expression status. Surgical specimen of ovarian cancer (A); biopsy specimen of ovarian cancer (B); endometrial cancer (C); cervical cancer (D). **Supplemental Fig. 2**. Changes in HER3 expression between primary and recurrent status. Proportion of HER3-high at primary and recurrent status in ovarian cancer; high-grade serous carcinomas vs. others (A), platinum-sensitive recurrence vs. resistant recurrence (B), localized relapse vs. distant metastases (C), and one type of prior therapy vs. two or more (D). Proportion of HER3-high at primary and recurrent status in endometrial cancer; low-grade vs. high-grade (E), localized relapse vs. distant metastases (F), and non-chemotherapy vs. prior chemotherapy (G). **Supplemental Fig. 3**. Kaplan‒Meier survival analysis of overall survival, including changed HER3 status at recurrence. (A) ovarian cancer and (B) endometrial cancer in patients with HER3-decreased vs. patients with HER3-no chang vs. patients with HER3-increased at recurrence.

## Data Availability

The data analyzed in this study are available from the corresponding author upon reasonable request.

## Abbreviations

HER: Human epidermal growth factor
EGFR: Epidermal growth factor receptor
CI: Confidence interval
HR: Hazard ratio
OS: Overall survival
IHC: Immunohistochemical
