# Supplementary material for: Changes in HER3 expression profiles between primary and recurrent gynecological cancers
Source: Cancer Cell Int. 2023 Feb 3;23:18. doi: 10.1186/s12935-022-02844-z (PMC9898949; doi:10.1186/s12935-022-02844-z)
Supplement: Supplementary file 1 — Additional file 1: Table S1. Patient characteristics of ovarian cancer [file 12935_2022_2844_MOESM1_ESM.docx]

Table S1. Patient characteristics of ovarian cancer

|  | **N = 40** | **％** |
| --- | --- | --- |
| **At initial diagnosis** |  | |
| Age (median, range) | 56 (36–76) | |
| Histology |  |  |
| High-grade serous carcinoma | 27 | 67.5 |
| Clear cell carcinoma | 4 | 10.0 |
| Endometrioid carcinoma | 5 | 12.5 |
| Mucinous carcinoma | 1 | 2.5 |
| Low-grade serous carcinoma | 2 | 5.0 |
| Others | 1 | 2.5 |
| Stage at initial diagnosis |  |  |
| I–II | 7 | 17.5 |
| III | 23 | 57.5 |
| IV | 10 | 25.0 |
| Prior neoadjuvant chemotherapy |  |  |
| Yes | 13 | 32.5 |
| No | 27 | 67.5 |
| **At recurrent status** |  |  |
| Number of previous chemotherapy regimens |  |  |
| 1 | 28 | 70.0 |
| 2 | 3 | 7.5 |
| 3 | 4 | 10.0 |
| >3 | 5 | 12.5 |
| Platinum sensitivity |  |  |
| Sensitive | 29 | 72.5 |
| Refractory | 11 | 27.5 |
| Prior radiotherapy |  |  |
| Yes | 1 | 2.5 |
| No | 39 | 97.5 |
| Site of a recurrence |  |  |
| Local | 16 | 40.0 |
| Metastatic | 24 | 60.0 |
