# Supplementary material for: Changes in HER3 expression profiles between primary and recurrent gynecological cancers
Source: Cancer Cell Int. 2023 Feb 3;23:18. doi: 10.1186/s12935-022-02844-z (PMC9898949; doi:10.1186/s12935-022-02844-z)
Supplement: Supplementary file 2 — Additional file 2: Table S2. Patient characteristics of endometrial cancer [file 12935_2022_2844_MOESM2_ESM.docx]

Table S2. Patient characteristics of endometrial cancer

|  | **N = 32** | **％** |
| --- | --- | --- |
| **At initial diagnosis** |  | |
| Age (median, range) | 64 (51–84) | |
| Histology |  |  |
| Endometrioid, G1 | 8 | 25.0 |
| Endometrioid, G2 | 7 | 21.9 |
| Endometrioid, G3 | 2 | 6.3 |
| Clear cell carcinoma | 2 | 6.3 |
| Serous carcinoma | 5 | 15.6 |
| Carcinosarcoma | 7 | 21.9 |
| Others | 1 | 3.1 |
| Stage at initial diagnosis |  |  |
| I–II | 11 | 34.4 |
| III | 17 | 53.1 |
| IV | 4 | 12.5 |
| **At recurrent status** |  |  |
| Number of previous chemotherapy regimens |  |  |
| 0 | 15 | 46.9 |
| 1 | 15 | 46.9 |
| 2 | 2 | 6.3 |
| Prior radiotherapy |  |  |
| Yes | 4 | 12.5 |
| No | 28 | 87.5 |
| Site of a recurrence |  |  |
| Local | 21 | 65.6 |
| Metastatic | 11 | 34.4 |
