# Supplementary material for: Changes in HER3 expression profiles between primary and recurrent gynecological cancers
Source: Cancer Cell Int. 2023 Feb 3;23:18. doi: 10.1186/s12935-022-02844-z (PMC9898949; doi:10.1186/s12935-022-02844-z)
Supplement: Supplementary file 3 — Additional file 3: Table S3. Patient characteristics of cervical cancer [file 12935_2022_2844_MOESM3_ESM.docx]

Table 3. Patient characteristics of cervical cancer

|  | **N = 14** | **％** |
| --- | --- | --- |
| **At initial diagnosis** |  | |
| Age (median, range) | 49 (31–63) | |
| Histology |  |  |
| SCC, keratinizing | 5 | 35.5 |
| SCC, non-keratinizing | 3 | 21.3 |
| Adenocarcinoma, endocervical type | 2 | 14.2 |
| Adenosquamous carcinoma | 1 | 7.1 |
| Adenocarcinoma, gastric-type | 2 | 14.2 |
| Others | 1 | 7.1 |
| Stage at initial diagnosis |  |  |
| I–II | 13 | 92.9 |
| III-IV | 1 | 7.1 |
| **At recurrent status** |  |  |
| Number of previous chemotherapy regimens |  |  |
| 0 | 12 | 85.7 |
| 1 | 2 | 14.3 |
| Prior radiotherapy |  |  |
| Yes | 8 | 57.1 |
| No | 6 | 42.9 |
| Site of a recurrence |  |  |
| Local | 8 | 57.1 |
| Metastatic | 6 | 42.9 |
