# Supplementary material for: Changes in HER3 expression profiles between primary and recurrent gynecological cancers
Source: Cancer Cell Int. 2023 Feb 3;23:18. doi: 10.1186/s12935-022-02844-z (PMC9898949; doi:10.1186/s12935-022-02844-z)
Supplement: Supplementary file 4 — Additional file 4: Table S4. HER3 expression in ovarian cancer at initial diagnosis [file 12935_2022_2844_MOESM4_ESM.docx]

Table S4. HER3 expression in ovarian cancer at initial diagnosis

| **HER3 score** | **0** | **1+** | **2+** | **3+** | **2+/3+** | **1+/2+/3+** |
| --- | --- | --- | --- | --- | --- | --- |
| High-grade serous carcinoma (N=27) | 1  (3.7%) | 9  (33.3%) | 6  (22.2%) | 11  (40.7%) | 17  (62.9%) | 26  (96.3%) |
| Clear cell carcinoma (N=4) | 0  (0.0%) | 1  (25.0%) | 0  (0.0%) | 3  (75.0%) | 3  (75.0%) | 4  (100.0%) |
| Endometrioid carcinoma (N=5) | 0  (0.0%) | 0  (0.0%) | 4  (80.0%) | 1  (20.0%) | 5  (100.0%) | 5  (100%) |
| Mucinous carcinoma (N=1) | 0  (0.0%) | 0  (0.0%) | 0  (0.0%) | 1  (100.0%) | 1  (100.0%) | 1  (100.0%) |
| Others (N=3) | 1  (33.3%) | 1  (33.3%) | 1  (33.3%) | 0  (0.0%) | 1  (33.3%) | 2  (66.7%) |
| Total (N=40) | 2  (5.0%) | 11  (27.5%) | 11  (27.5%) | 16  (40.0%) | 27  (67.5%) | 38  (95.0%) |
