# Supplementary material for: Changes in HER3 expression profiles between primary and recurrent gynecological cancers
Source: Cancer Cell Int. 2023 Feb 3;23:18. doi: 10.1186/s12935-022-02844-z (PMC9898949; doi:10.1186/s12935-022-02844-z)
Supplement: Supplementary file 5 — Additional file 5: Table S5. HER3 expression in endometrial cancer at initial diagnosis [file 12935_2022_2844_MOESM5_ESM.docx]

Table S5. HER3 expression in endometrial cancer at initial diagnosis

| **HER3 score** | **0** | **1+** | **2+** | **3+** | **2+/3+** | **1+/2+/3+** |
| --- | --- | --- | --- | --- | --- | --- |
| Endometrioid, G1 and G2 (N=15) | 4  (26.7%) | 9  (60.0%) | 1  (6.7%) | 1  (6.7%) | 2  (13.4%) | 11  (73.4%) |
| Endometrioid, G3 (N=2) | 0  (0%) | 1  (50.0%) | 1  (50.0%) | 0  (0.0%) | 1  (50.0%) | 2  (100%) |
| Clear cell carcinoma (N=2) | 0  (0%) | 0  (0.0%) | 0  (0%) | 2  (100%) | 2  (100%) | 2  (100%) |
| Serous carcinoma (N=5) | 0  (0.0%) | 1  (20.0%) | 2  (40.0%) | 2  (40.0%) | 4  (80.0%) | 5  (100%) |
| Carcinosarcoma (N=7) | 0  (0.0%) | 2  (28.6%) | 3  (42.9%) | 2  (28.6%) | 5  (71.4%) | 7  (100.0%) |
| Others (N=1) | 0  (0.0%) | 0  (0.0%) | 0  (0.0%) | 1  (100.0%) | 1  (100.0%) | 1  (100.0%) |
| Total (N=32) | 4  (12.5%) | 13  (40.6%) | 7  (21.9%) | 8  (25.0%) | 15  (46.9%) | 28  (90.6%) |
