# Supplementary material for: Changes in HER3 expression profiles between primary and recurrent gynecological cancers
Source: Cancer Cell Int. 2023 Feb 3;23:18. doi: 10.1186/s12935-022-02844-z (PMC9898949; doi:10.1186/s12935-022-02844-z)
Supplement: Supplementary file 6 — Additional file 6: Table S6. HER3 expression in cervical cancer at initial diagnosis [file 12935_2022_2844_MOESM6_ESM.docx]

Table S6. HER3 expression in cervical cancer at initial diagnosis

| **HER3 score** | **0** | **1+** | **2+** | **3+** | **2+/3+** | **1+/2+/3+** |
| --- | --- | --- | --- | --- | --- | --- |
| Squamous cell carcinoma (N=8) | 0  (0.0%) | 1  (12.5%) | 4  (50.0%) | 3  (37.5%) | 7  (87.5%) | 8  (100.0%) |
| Adenocarcinoma, endocervical type (N=2) | 0  (0%) | 1  (50.0%) | 0  (0%) | 1  (50.0%) | 1  (50.0%) | 2  (100%) |
| Adenocarcinoma, gastric-type (N=2) | 0  (0%) | 0  (0.0%) | 0  (0%) | 2  (100%) | 2  (100%) | 2  (100%) |
| Adenosquamous carcinioma (N=1) | 0  (0.0%) | 0  (0.0%) | 1  (100.0%) | 0  (0.0%) | 1  (100.0%) | 1  (100.0%) |
| Others (N=1) | 0  (0.0%) | 0  (0.0%) | 0  (0.0%) | 1  (100.0%) | 1  (100.0%) | 1  (100.0%) |
| Total (N=14) | 0  (0.0%) | 2  (14.3%) | 5  (35.5%) | 7  (50.0%) | 12  (85.7%) | 14  (100.0%) |
