# Supplementary figures and images for: Changes in HER3 expression profiles between primary and recurrent gynecological cancers
Source: Cancer Cell Int. 2023 Feb 3;23:18. doi: 10.1186/s12935-022-02844-z (PMC9898949; doi:10.1186/s12935-022-02844-z)

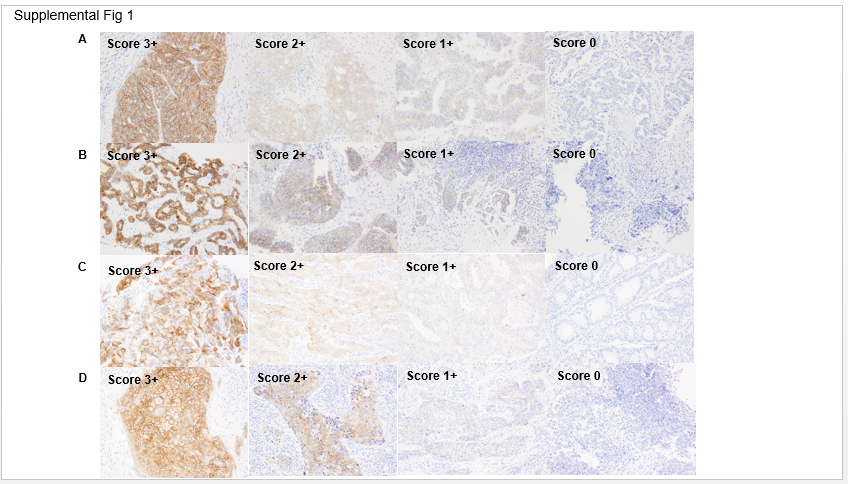


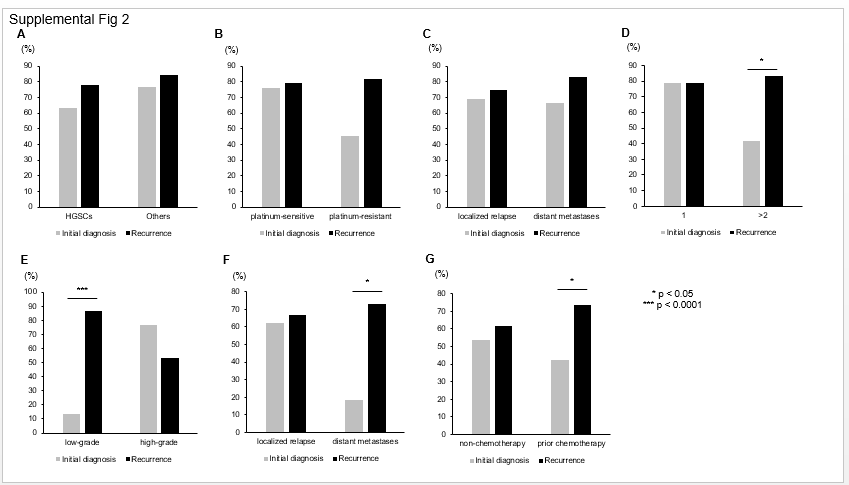


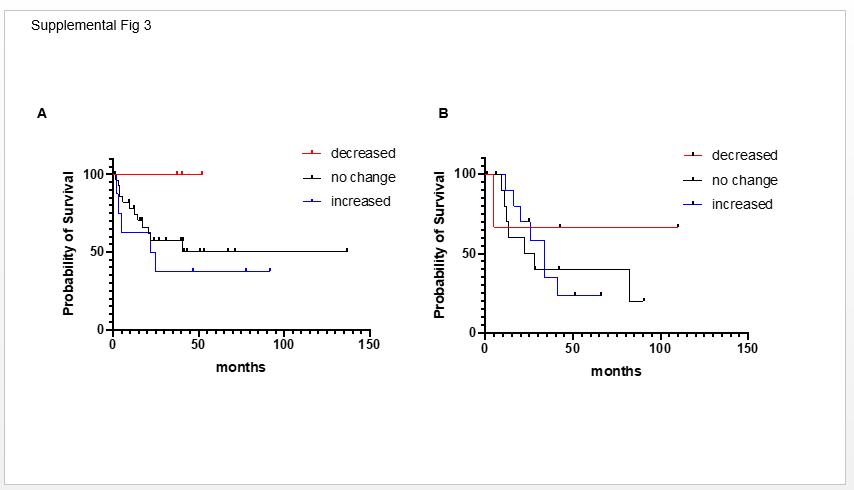

Supplement: Supplementary file 7 — Additional file 7. Supplemental Figs. 1–3. Supplemental Fig. 1. Histological findings of HER3 expression status. Surgical specimen of ovarian cancer (A); biopsy specimen of ovarian cancer (B); endometrial cancer (C); cervical cancer (D). Supplemental Fig. 2. Changes in HER3 expression between primary and recurrent status. Proportion of HER3-high at primary and recurrent status in ovarian cancer; high-grade serous carcinomas vs. others (A), platinum-sensitive recurrence vs. resistant recurrence (B), localized relapse vs. distant metastases (C), and one type of prior therapy vs. two or more (D). Proportion of HER3-high at primary and recurrent status in endometrial cancer; low-grade vs. high-grade (E), localized relapse vs. distant metastases (F), and non-chemotherapy vs. prior chemotherapy (G). Supplemental Fig. 3. Kaplan‒Meier survival analysis of overall survival, including changed HER3 status at recurrence. (A) ovarian cancer and (B) endometrial cancer in patients with HER3-decreased vs. patients with HER3-no chang vs. patients with HER3-increased at recurrence. [file 12935_2022_2844_MOESM7_ESM.docx]
